# Supplementary material for: Longitudinal multicompartment characterization of host-microbiota interactions in patients with acute respiratory failure
Source: Nat Commun. 2024 Jun 3;15:4708. doi: 10.1038/s41467-024-48819-8 (PMC11148165; doi:10.1038/s41467-024-48819-8)
Supplement: Supplementary file 1 — Supplementary Information [file 41467_2024_48819_MOESM1_ESM.pdf]

Supplementary Information

Table S1: Clinical characteristics of subjects enrolled in the COVID-19 cohorts.

	UPMC-COVID	MGH-COVID
N	49	97
Age, years (median [IQR])	63.0 [57.1, 71.7]	63.0 [51.0, 71.0]
Men, n (%)	30 (61.2)	54 (55.7)
Whites, n (%)	37 (75.5)	70 (72.2)
BMI (median [IQR])	33.9 [29.0, 41.8]	30.1 [26.6, 36.0]
COPD, n (%)	10 (20.4)	38 (39.2)
Diabetes, n (%)	21 (42.9)	30 (30.9)
WBC (median [IQR])	11.2 [8.6, 14.1]	NA
Creatinine (median [IQR])	1.0 [0.7, 1.6]	NA
Plateau Pressure (median [IQR])	26.0 [20.0, 29.0]	NA
PaO2:FiO2 ratio (median [IQR])	94.0 [69.8, 168.2]	NA
Hypoinflammatory subphenotype, n (%)	42 (87.5)	NA
VFD (median [IQR])	0.0 [0.0, 13.0]	NA
Severe Disease, n (%)	49 (100)	41 (42.3)
Mortality 60-day, n (%)	23 (46.9)	10 (10.3)

Source data are provided as a Source Data file.

Figure S1:

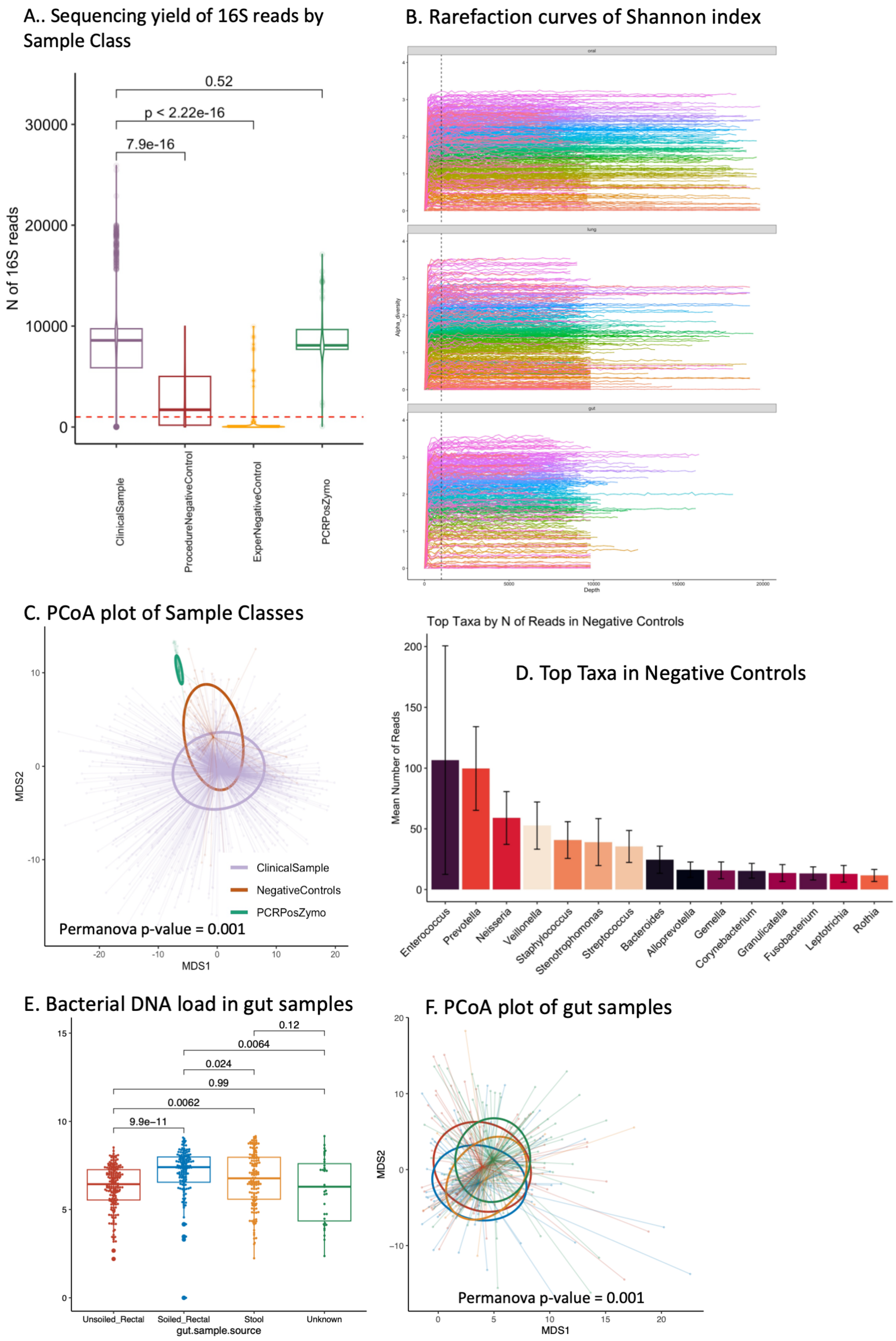

A.. Sequencing yield of 16S reads by Sample Class
N of 16S reads
0.52
p < 2.22e-16
7.9e-16
ClinicalSample
ProcedureNegativeControl
ExperNegativeControl
PCRPosZymo
B. Rarefaction curves of Shannon index
oral
lung
gut
Alpha_diversity
Depth
C. PCoA plot of Sample Classes
MDS1
MDS2
ClinicalSample
NegativeControls
PCRPosZymo
Permanova p-value = 0.001
Top Taxa by N of Reads in Negative Controls
D. Top Taxa in Negative Controls
Mean Number of Reads
Enterococcus
Prevotella
Neisseria
Veillonella
Staphylococcus
Stenotrophomonas
Streptococcus
Bacteroides
Alloprevotella
Gemella
Corynebacterium
Granulicatella
Fusobacterium
Leptotrichia
Rothia
E. Bacterial DNA load in gut samples
0.12
0.0064
0.024
0.99
0.0062
9.9e−11
Unsoiled_Rectal
Soiled_Rectal
Stool
Unknown
gut.sample.source
F. PCoA plot of gut samples
MDS1
MDS2
Permanova p-value = 0.001

Quality control steps for clinical and experimental control samples by Illumina MiSeq 16S rRNA gene sequencing. A. Clinical samples from ICU patients and healthy control subjects had much higher 16S-Seq yield of high-quality reads compared to experimental and procedure negative controls. Red dashed line at 1,000 reads. B. Shannon index (alpha diversity) rarefaction curves by sequencing depth for the clinical samples from ICU patients (oral, lung, and gut), illustrating that all samples reached plateau of Shannon indices at the cut-off of 1,000 reads used in analyses. C. Clinical samples had markedly different bacterial composition based on Centered-Log-Ratio (CLR)-transformed abundances of bacterial genera, shown as Bray-Curtis indices in Principal Coordinates Analysis (PCoA) compared to negative controls or PCR positive samples (Zymo Mock community controls), and analyzed by permutational analysis of variance (Permanova). D. Top taxa detected in negative control samples, indicating possible contamination. Overall, such taxa were detected at very low levels (mean number of reads <100). E-F. Quality control examination for gut samples. Unsoiled rectal swabs (i.e. not visibly coated by stool) had markedly lower bacterial burden (examined by qPCR of 16S rRNA gene) and differential composition compared to soiled rectal swabs or stool samples, and therefore, unsoiled rectal swabs were excluded from further analysis as they may not provide sufficient representation of gut microbiota. Source data are provided as a Source Data file. Data displayed as boxplots with individual dots have their median as the line inside the box, interquartile range (25th-75th percentile) as the box itself, whiskers extend to 1.5 times the interquartile range, and individual dots beyond whiskers signify outlier observations.

Figure S2:

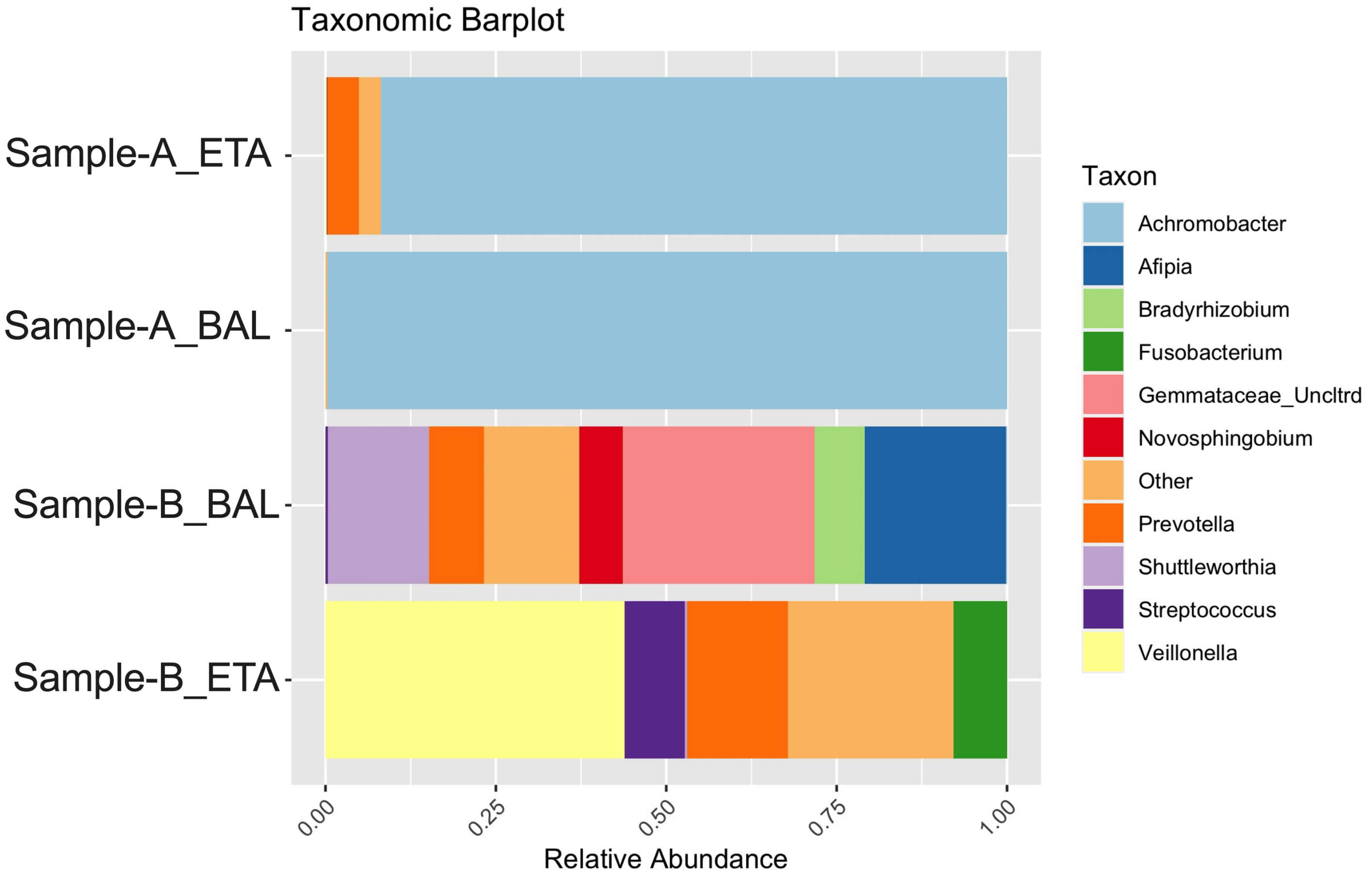

Taxonomic comparison of top 10 abundant taxa between Endotracheal Aspirate (ETA) and Bronchoalveolar Lavage (BAL) samples that were synchronously obtained from the same subject. For subject A, both ETA and BAL samples showed near complete community dominance by Achromobacter genera (>90%). Subject A was clinically diagnosed with Achromobacter xylosidans pneumonia based on BAL microbiologic cultures, consistent with 16S-Seq data by both ETA and BAL. For subject B that had higher alpha diversity than subject A, there was limited taxonomic concordance between ETA and BAL sample, with several different top abundant taxa between sample types (top abundant taxon *Veillonella* in the ETA sample vs. *Gemmataceae* taxa in the BAL sample). Subject B had no bacterial growth in clinical BAL cultures and was diagnosed as culture-negative pneumonia, a diagnosis that was not supported by 16S-Seq of ETA or BAL sample. Source data are provided as a Source Data file.

Figure S3.

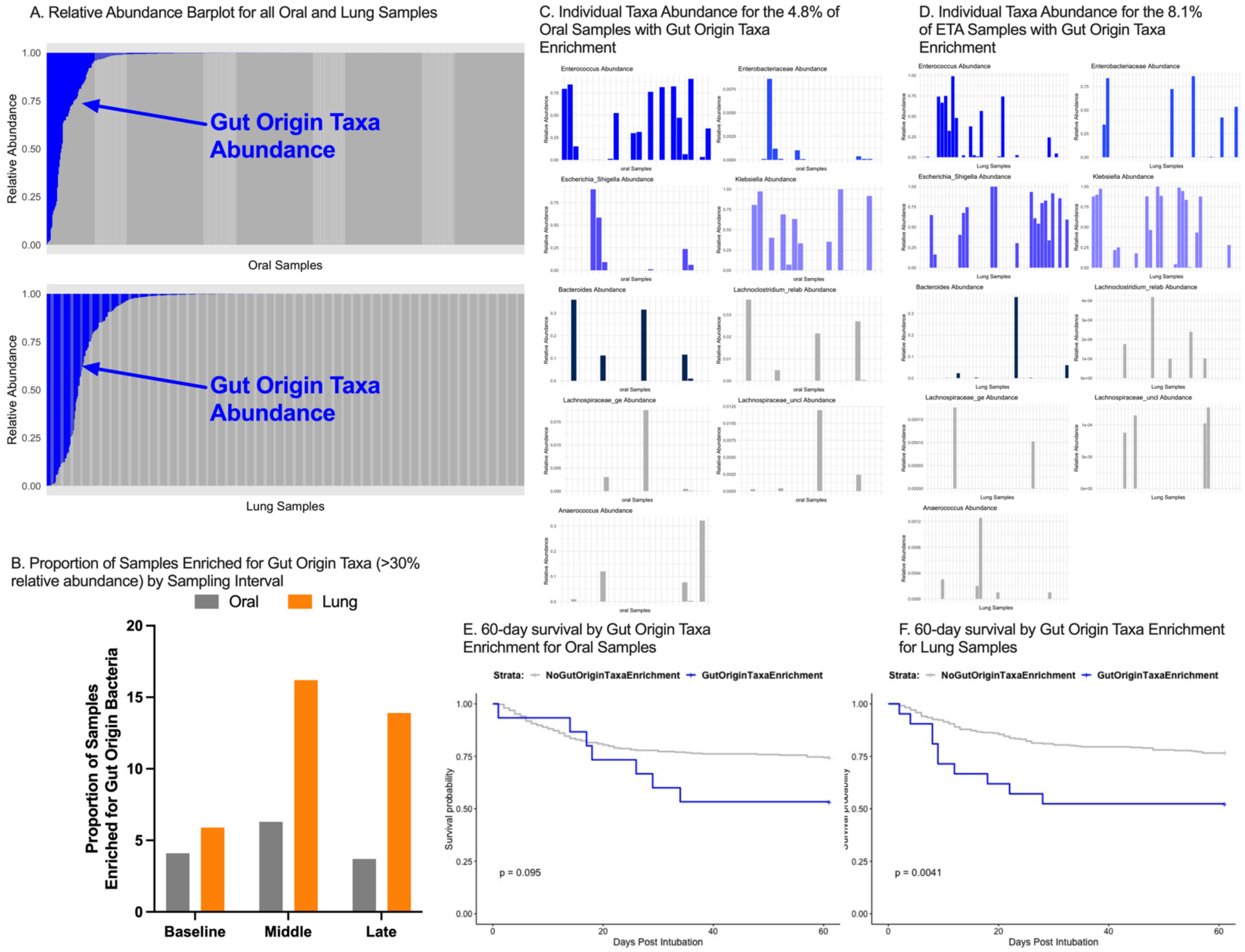

Enrichment of respiratory track samples by gut origin bacteria. A. Relative abundance of all available oral and lung samples for gut origin taxa (*Enterococcus, Enterobacteriaceae, Escherichia_Shigella, Klebsiella, Bacteroides, Lachonoclostridium, Lachnospiraceae_ge, Lachnospiraceae_uncl, Anaerococcus*) vs. other taxa. 4.5% and 8.3% of all oral and lung samples, respectively, had >30% relative abundance for gut-origin taxa (Fisher's p=0.03), classified as samples with Gut Enrichment. B. Proportion of oral lung samples with gut origin taxa enrichment across the three time intervals of sampling. There was increase in the proportion of lung samples with gut origin taxa enrichment from baseline to middle interval (Fisher test p=0.02). C-D. Relative abundance of individual gut origin taxa for the oral and lung samples with gut enrichment. Gut enrichment was mostly accounted for by organisms with pathogenic potential (*Enterococcus, Enterobacteriaceae, Escherichia_Shigella* and *Klebsiella*, shown in blue colors) than gut origin commensals (*Bacteroides, Lachonoclostridium, Lachnospiraceae_ge, Lachnospiraceae_uncl, Anaerococcus*). E-F. Patients with gut origin

taxa enrichment in lung samples at baseline had significantly worse 60-day survival (log rank p=0.004) compared to patients without enrichment for gut origin taxa, whereas gut enrichment in oral samples did not impact survival. Source data are provided as a Source Data file.

Table S2: Classification of analyzed taxa by oxygen requirements.

Oxygen Requirement	Taxa
Aerobe	Acinetobacter, Afipia, Bergeyella, Bradyrhizobium, Cardiobacterium, Comamonas, Methylobacterium_Methylorubrum, Neisseria, Paucibacter, Pseudomonadaceae_uncl, Pseudomonas, Ralstonia, Salinicoccus, Sphingomonas, Stenotrophomonas
Obligate Anaerobes	Acidaminococcus, Agathobacter, Alistipes, Alloprevotella, Alloscardovia, Anaerococcus, Anaeroglobus, Anaerostipes, Anaerotruncus, Anaerovoracaceae_ge, Anaerovoracaceae_uncl, Anaerovoracaceae_Uncltrd, Bacteroidales_uncl, Bacteroides, Bifidobacterium, Bilophila, Blautia, Bulleidia, Butyricicoccus, Butyricimonas, Butyrivibrio, Catonella, Centipeda, Christensenellaceae_R_7_grp, Christensenellaceae_Uncltrd, Clostridia_UCG_014_ge, Clostridia_vadinBB60_grp_ge, Clostridium_sensu_stricto_1, Collinsella, Coprococcus, Cryptobacterium, Desulfovibrio, Dorea, Eggerthella, Eisenbergiella, Erysipelatoclostridium, Eubacterium, Ezakiella, F0058, Faecalibacterium, Faecalitalea, Fastidiosipila, Fenollaria, Filifactor, Finegoldia, Flavonifractor, Fretibacterium, Fusicatenibacter, Fusobacterium, Howardella, Hungatella, Intestinibacter, Intestinimonas, Johnsonella, Lachnoanaerobaculum, Lachnoclostridium, Lachnospiraceae_ge, Lachnospiraceae_UCG_010, Lachnospiraceae_uncl, Lachnospiraceae_Uncltrd, Lawsonella, Lentimicrobium, Megasphaera, Methanobrevibacter, Mobiluncus, Mogibacterium, Monoglobus, Murdochiella, Negativicoccus, Odoribacter, Olsenella, Oribacterium, Oscillibacter, Oscillospirales_ge, Parabacteroides, Parascardovia, Parasutterella, Parvimonas, Peptococcus, Peptoniphilus, Peptostreptococcaceae_ge, Peptostreptococcus, Phascolarctobacterium, Phocaeicola, Porphyromonas, Prevotella, Prevotellaceae_UCG_001, Prevotellaceae_uncl, Prevotellaceae_Uncltrd, Prevotellaceae_YAB2003_grp, Pseudoramibacter, Rikenellaceae_RC9_gut_grp, Romboutsia, Roseburia, Ruminococcaceae_IncSed, Ruminococcaceae_uncl, Ruminococcus, Scardovia, Sellimonas, Shuttleworthia, Slackia, Solobacterium, Stomatobaculum, Sutterella, Tannerella, Tannerellaceae_uncl, Veillonella, Veillonellaceae_ge, Veillonellaceae_uncl
Facultative Anaerobes	Citrobacter, Klebsiella, Lactococcus, Abiotrophia, Actinobacillus, Actinomyces, Aerococcaceae_uncl, Aggregatibacter, Capnocytophaga, Dolosigranulum, Eikenella, Enterobacterales_uncl, Enterobacteriaceae_uncl, Enterococcus, Escherichia_Shigella, Facklamia, Gardnerella, Gemella, Granulicatella, Haemophilus, Lactobacillus, Lautropia, Listeria, Pasteurellaceae_uncl, Providencia, Pseudocitrobacter, Saccharimonadaceae_ge, Saccharimonadales_ge, Salmonella, Staphylococcaceae_uncl, Staphylococcus, Streptococcaceae_uncl, Streptococcus, Varibaculum
Microaerophile	Akkermansia, Aquabacterium, Campylobacter
Unclassifiable	Absconditabacteriales_SR1_ge, Actinobacteria_uncl, Alcaligenaceae_ge, Bacillaceae_uncl, Bacilli_uncl, Bacillus, Bacteria_uncl, Bacteroidia_uncl, Carnobacteriaceae_uncl, Chloroplast_ge, Clostridia_uncl, Colidextribacter, Conchiformibius, Defluviitaleaceae_UCG_011, DTU089, Family_XIII_AD3011_grp, Family_XIII_UCG_001, Lactobacillales_uncl, Micrococcales_uncl, Mitochondria_ge, Muribaculaceae_ge, Negativibacillus, NK4A214_grp, Oscillospiraceae_uncl, Oscillospiraceae_Uncltrd, Oscillospirales_uncl, S5_A14a, Ruminococcaceae_Uncltrd, Selenomonadaceae_uncl, Subdoligranulum, TM7x, Tuzzerella, UBA1819, UCG_002, UCG_005, Veillonellales_Selenomonadales_uncl, W5053
Variable	Atopobium, Bacillales_uncl, Bifidobacteriaceae_uncl, Corynebacterium, Dialister, Erysipelotrichaceae_ge, F0332, Firmicutes_uncl, Gammaproteobacteria_uncl, Kingella, Leptotrichia, Mycoplasma, Neisseriaceae_uncl, Rothia, Selenomonas, Treponema

Table S3: Classification of analyzed taxa by plausible pathogenicity for lung infections.

Pathogenicity Classification	Taxa
Oral Commensals	Alloprevotella, Fusobacterium, Gemella, Granulicatella, Haemophilus, Leptotrichia, Neisseria, Prevotella, Prevotellaceae_UCG_001, Prevotellaceae_uncl, Prevotellaceae_Uncltrd, Prevotellaceae_YAB2003_grp, Rothia, Selenomonas, Streptococcus, Veillonella, Veillonellaceae_ge, Veillonellaceae_uncl
Respiratory Pathogens	Acinetobacter, Citrobacter, Eikenella, Enterobacteriaceae_uncl, Enterococcus, Escherichia_Shigella, Klebsiella, Listeria, Pseudomonadaceae_uncl, Pseudomonas, Staphylococcus, Stenotrophomonas
Other	Abiotrophia, Absconditabacteriales_SR1_ge, Acidaminococcus, Actinobacillus, Actinobacteria_uncl, Actinomyces, Aerococcaceae_uncl, Afipia, Agathobacter, Aggregatibacter, Akkermansia, Alcaligenaceae_ge, Alistipes, Alloscardovia, Anaerococcus, Anaeroglobus, Anaerostipes, Anaerotruncus, Anaerovoracaceae_ge, Anaerovoracaceae_uncl, Anaerovoracaceae_Uncltrd, Aquabacterium, Atopobium, Bacillaceae_uncl, Bacillales_uncl, Bacilli_uncl, Bacillus, Bacteria_uncl, Bacteroidales_uncl, Bacteroides, Bacteroidia_uncl, Bergeyella, Bifidobacteriaceae_uncl, Bifidobacterium, Bilophila, Blautia, Bradyrhizobium, Bulleidia, Butyricicoccus, Butyricimonas, Butyrivibrio, Campylobacter, Capnocytophaga, Cardiobacterium, Carnobacteriaceae_uncl, Catonella, Centipeda, Chloroplast_ge, Christensenellaceae_R_7_grp, Christensenellaceae_Uncltrd, Clostridia_UCG_014_ge, Clostridia_uncl, Clostridia_vadinBB60_grp_ge, Clostridium_sensu_stricto_1, Colidextribacter, Collinsella, Comamonas, Conchiformibius, Coprococcus, Corynebacterium, Cryptobacterium, Defluviitaleaceae_UCG_011, Desulfovibrio, Dialister, Dolosigranulum, Dorea, DTU089, Eggerthella, Eisenbergiella, Enterobacterales_uncl, Erysipelatoclostridium, Erysipelotrichaceae_ge, Eubacterium, Ezakiella, F0058, F0332, Facklamia, Faecalibacterium, Faecalitalea, Family_XIII_AD3011_grp, Family_XIII_UCG_001, Fastidiosipila, Fenollaria, Filifactor, Finegoldia, Firmicutes_uncl, Flavonifractor, Fretibacterium, Fusicatenibacter, Gammaproteobacteria_uncl, Gardnerella, Howardella, Hungatella, Intestinibacter, Intestinimonas, Johnsonella, Kingella, Lachnoanaerobaculum, Lachnoclostridium, Lachnospiraceae_ge, Lachnospiraceae_UCG_010, Lachnospiraceae_uncl, Lachnospiraceae_Uncltrd, Lactobacillales_uncl, Lactobacillus, Lactococcus, Lautropia, Lawsonella, Lentimicrobium, Megasphaera, Methanobrevibacter, Methylobacterium_Methylorubrum, Micrococcales_uncl, Mitochondria_ge, Mobiluncus, Mogibacterium, Monoglobus, Murdochiella, Muribaculaceae_ge, Mycoplasma, Negativibacillus, Negativicoccus, Neisseriaceae_uncl, NK4A214_grp, Odoribacter, Olsenella, Oribacterium, Oscillibacter, Oscillospiraceae_uncl, Oscillospiraceae_Uncltrd, Oscillospirales_ge, Oscillospirales_uncl, Parabacteroides, Parascardovia, Parasutterella, Parvimonas, Pasteurellaceae_uncl, Paucibacter, Peptococcus, Peptoniphilus, Peptostreptococcaceae_ge, Peptostreptococcus, Phascolarctobacterium, Phocaeicola, Porphyromonas, Providencia, Pseudocitrobacter, Pseudoramibacter, Ralstonia, Rikenellaceae_RC9_gut_grp, Romboutsia, Roseburia, Ruminococcaceae_IncSed, Ruminococcaceae_uncl, Ruminococcaceae_Uncltrd, Ruminococcus, S5_A14a, Saccharimonadaceae_ge, Saccharimonadales_ge, Salinicoccus, Salmonella, Scardovia, Selenomonadaceae_uncl, Sellimonas, Shuttleworthia, Slackia, Solobacterium, Sphingomonas, Staphylococcaceae_uncl, Stomatobaculum, Streptococcaceae_uncl, Subdoligranulum, Sutterella, Tannerella, Tannerellaceae_uncl, TM7x, Treponema, Tuzzerella, UBA1819, UCG_002, UCG_005, Varibaculum, Veillonellales_Selenomonadales_uncl, W5053

Figure S4:

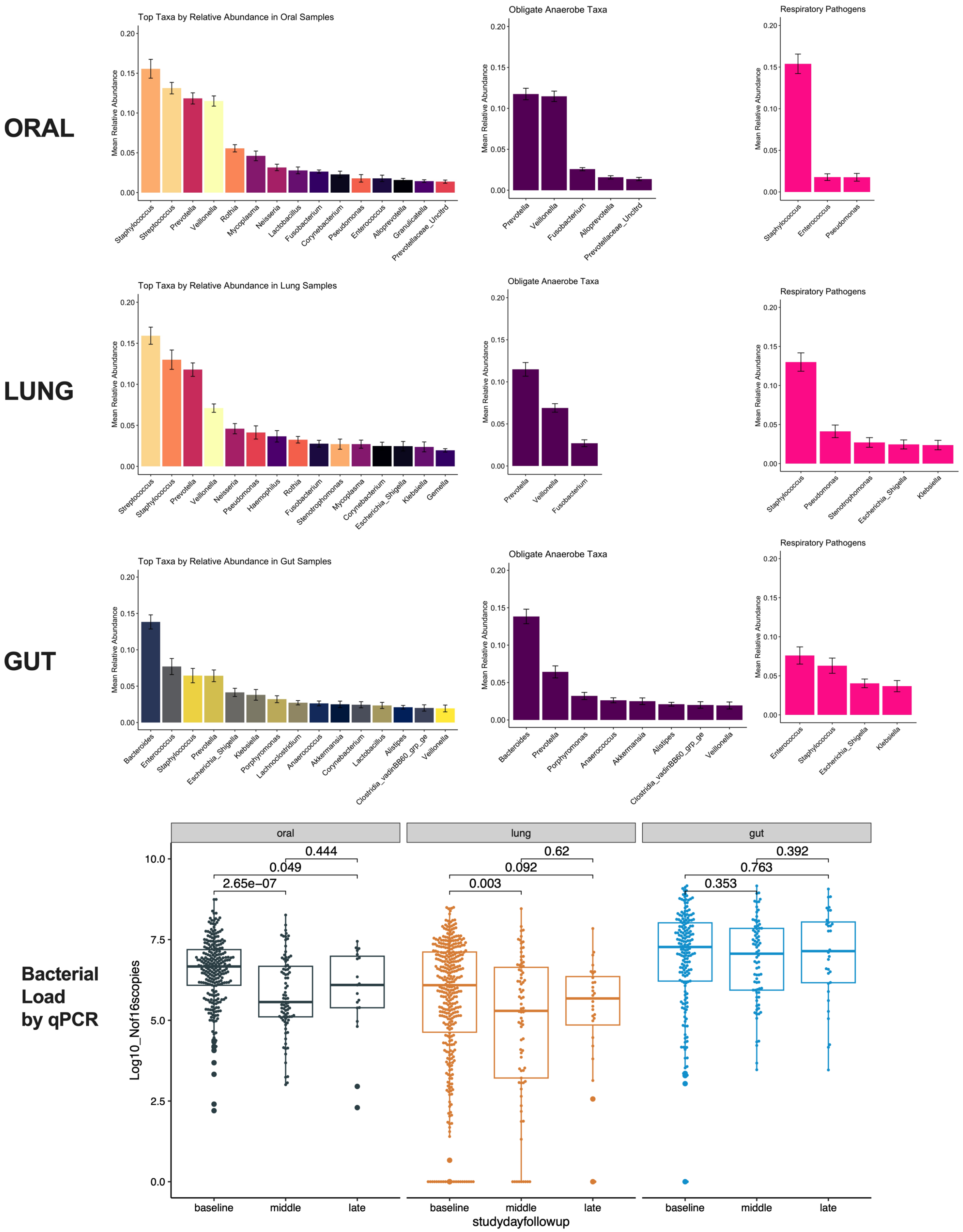

Top 15 representative taxa in each body compartment (oral, lung and gut). Each taxon is shown by barplots representing the mean relative abundance with the associated standard errors. Members of these top taxa classified as obligate anaerobes (middle panels) or as respiratory pathogens (right panels). The bottom panel displays the longitudinal measurement of bacterial load by 16S qPCR in each compartment. Source data

are provided as a Source Data file. Data displayed as boxplots with individual dots have their median as the line inside the box, interquartile range (25th-75th percentile) as the box itself, whiskers extend to 1.5 times the interquartile range, and individual dots beyond whiskers signify outlier observations.

Figure S5:

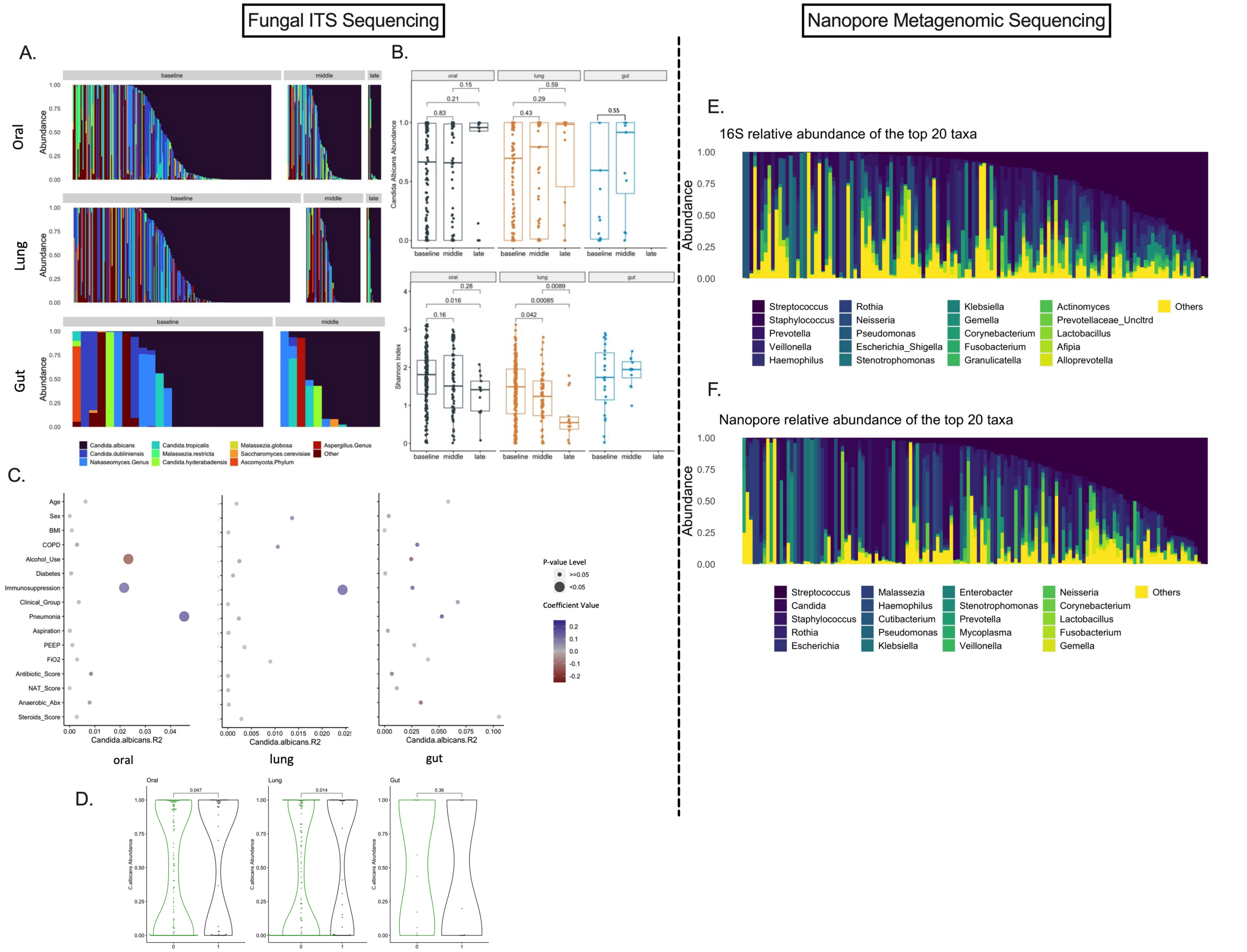

Fungal ITS sequencing and Nanopore metagenomic results. ITS sequencing was performed in samples from all three compartments [oral (n=226), lung (n=287), gut (n=31)], whereas Nanopore metagenomics was performed in 130 lung samples. *C.albicans* was the most abundant fungus and dominated (>50% relative abundance) more than half of samples of all three compartments at all time points (A-B). Oral and lung communities in follow-up samples had lower fungal Shannon index compared to baseline samples (B). History of immunosuppression and diagnosis of pneumonia were positively correlated with *C.albicans* abundance in oral and lung samples (C). D. Non-survivors had higher *C.albicans* abundance in oral and lung samples compared to survivors. E-F. Comparison of 16S and Nanopore derived lung communities showed that *Streptococcus* was the most abundant taxon by both methodologies, whereas Nanopore metagenomics confirmed that *C.albicans* was the second most abundant taxon in lung communities. Source data are provided

as a Source Data file. Data displayed as boxplots with individual dots have their median as the line inside the box, interquartile range (25th-75th percentile) as the box itself, whiskers extend to 1.5 times the interquartile range, and individual dots beyond whiskers signify outlier observations.

Figure S6:

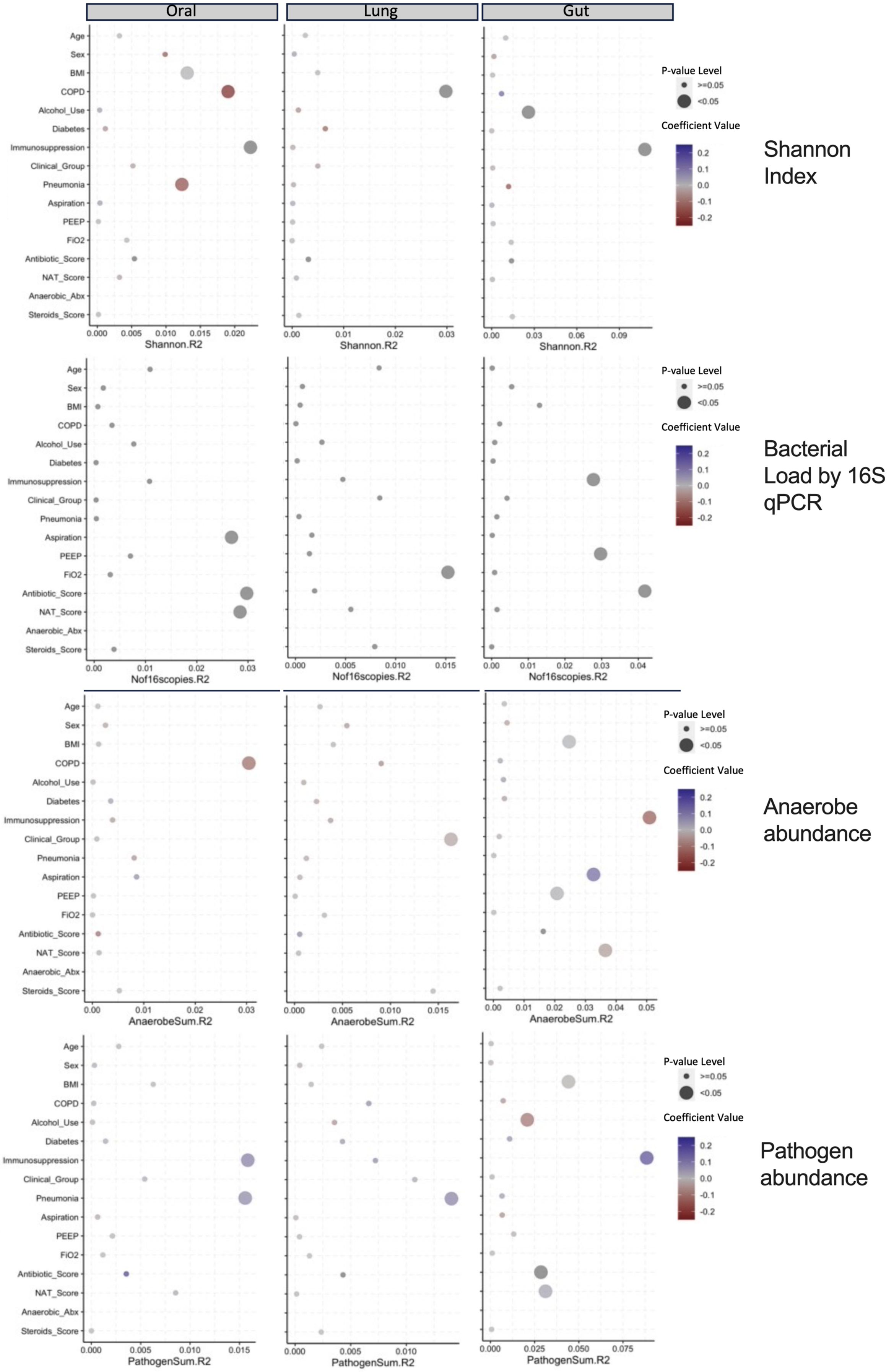
Oral
Lung
Gut
Age
Sex
BMI
COPD
Alcohol_Use
Diabetes
Immunosuppression
Clinical_Group
Pneumonia
Aspiration
PEEP
FiO2
Antibiotic_Score
NAT_Score
Anaerobic_Abx
Steroids_Score
Shannon.R2
Nof16scopies.R2
AnaerobeSum.R2
PathogenSum.R2
P-value Level
>=0.05
<0.05
Coefficient Value
0.2
0.1
0.0
-0.1
-0.2
Shannon Index
Bacterial Load by 16S qPCR
Anaerobe abundance
Pathogen abundance

Clinical variables associated with alpha diversity (Shannon Index), bacterial load (by 16S qPCR), obligate anaerobe and respiratory pathogen abundance in baseline samples from the three body compartments. Clinical variables are shown on the y-axis, and R^2 of linear regression models for Shannon index (obtained post rarefaction), bacterial load, anaerobe abundance (CLR-transformed), and pathogen abundance (CLR-transformed)) are shown on the x-axis. Statistically significant associations ($p<0.05$) are shown with large bubbles and direction of association is color coded. Source data are provided as a Source Data file.

Table S4: Mixed linear regression models for the examination of the effects of antibiotics and steroids on features of dysbiosis in samples from all three compartments. We examined the antibiotic exposure coded in three different ways: i) anaerobic coverage, ii) a numerical scale that included duration, timing and type, and iii) the Narrow Antibiotic Treatment (NAT) score. Each effect was adjusted for the study day from enrollment. The p-values of the mixed effects models with random patient intercepts are shown for each endpoint (columns) and significant values are highlighted in bold. Source data are provided as a Source Data file.

**Variables**	**Shannon index**	**Bacterial load (16S qPCR)**	**Obligate anaerobe abundance**	**Respiratory Pathogen abundance**
**Oral**				
Anaerobic_spectrum	0.422	**0.054**	**0.023**	0.526
Antibiotic_score	0.535	**0.010**	0.857	0.607
NAT_score	0.248	**0.006**	0.262	0.075
Steroids_score	0.499	0.793	0.542	0.116
**Lung**				
Anaerobic_spectrum	0.645	0.147	**0.037**	0.756
Antibiotic_score	0.605	0.841	0.578	0.842
NAT_score	0.456	0.064	0.734	0.262
Steroids_score	0.862	0.593	0.400	0.782
**Gut**				
Anaerobic_spectrum	0.348	0.801	**0.002**	**0.016**
Antibiotic_score	0.067	**0.006**	**0.004**	**0.031**
NAT_score	0.590	0.883	**0.008**	**0.040**
Steroids_score	0.671	0.437	0.083	0.218

Figure S7:

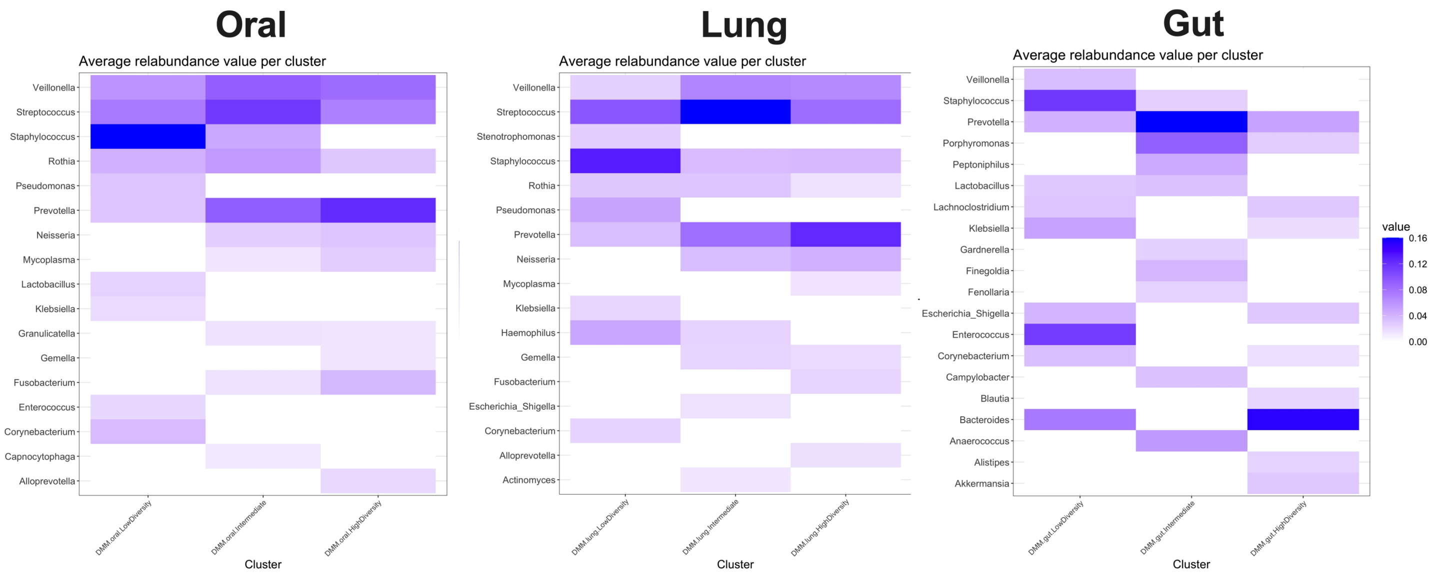

Heatmaps of relative abundance for the top 15 taxa in each compartment grouped by bacterial DMM clusters. Source data are provided as a Source Data file.

Figure S8:

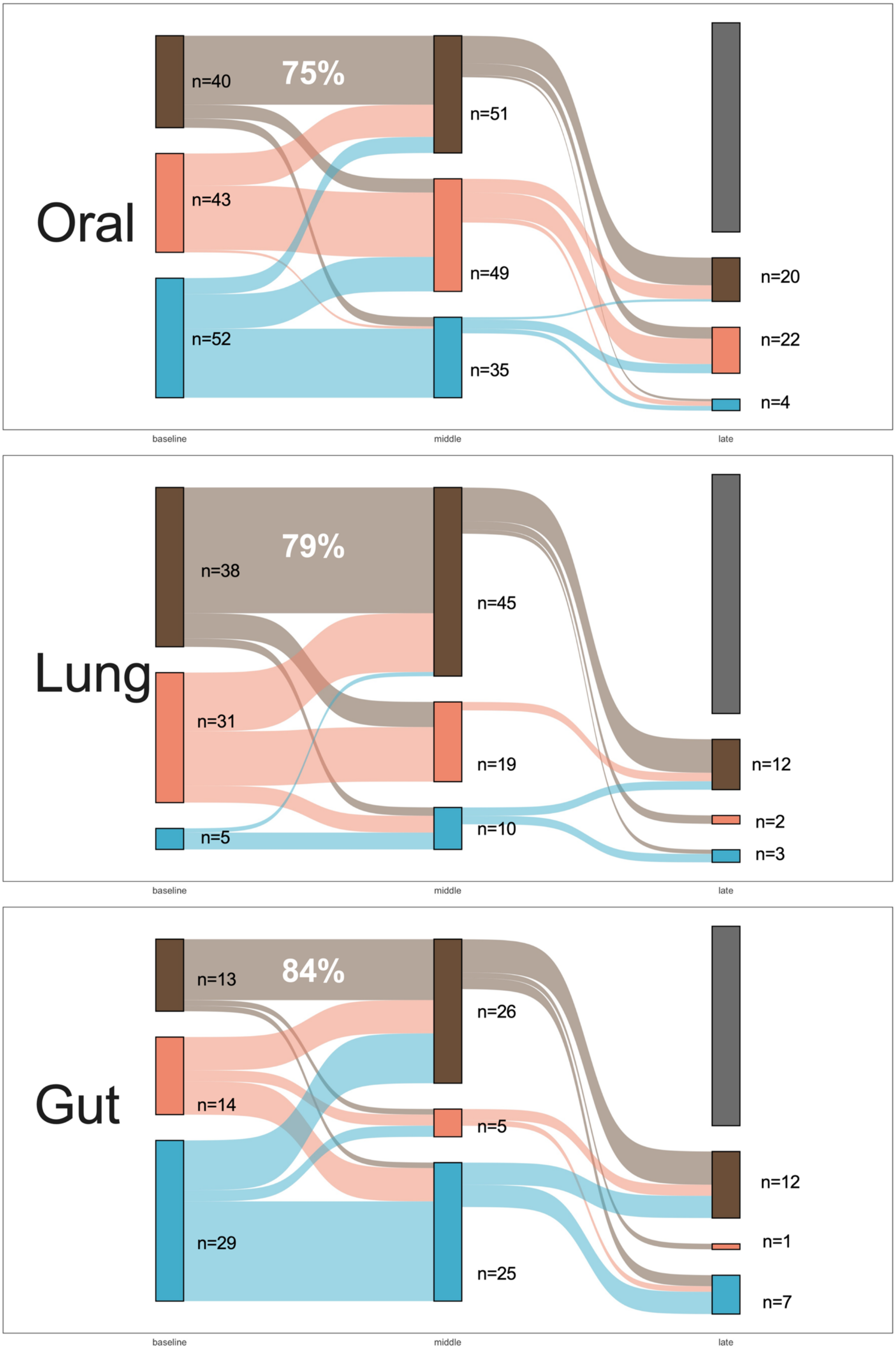

Longitudinal analysis of bacterial DMM cluster assignments showed overall stability from baseline to middle interval for Low-Diversity samples in each compartment. Low-Diversity cluster is shown in brown,

Intermediate-Diversity in light red, and High-Diversity in blue. Only subjects with available samples on both baseline and middle intervals are included in this analysis. Source data are provided as a Source Data file.

Figure S9.

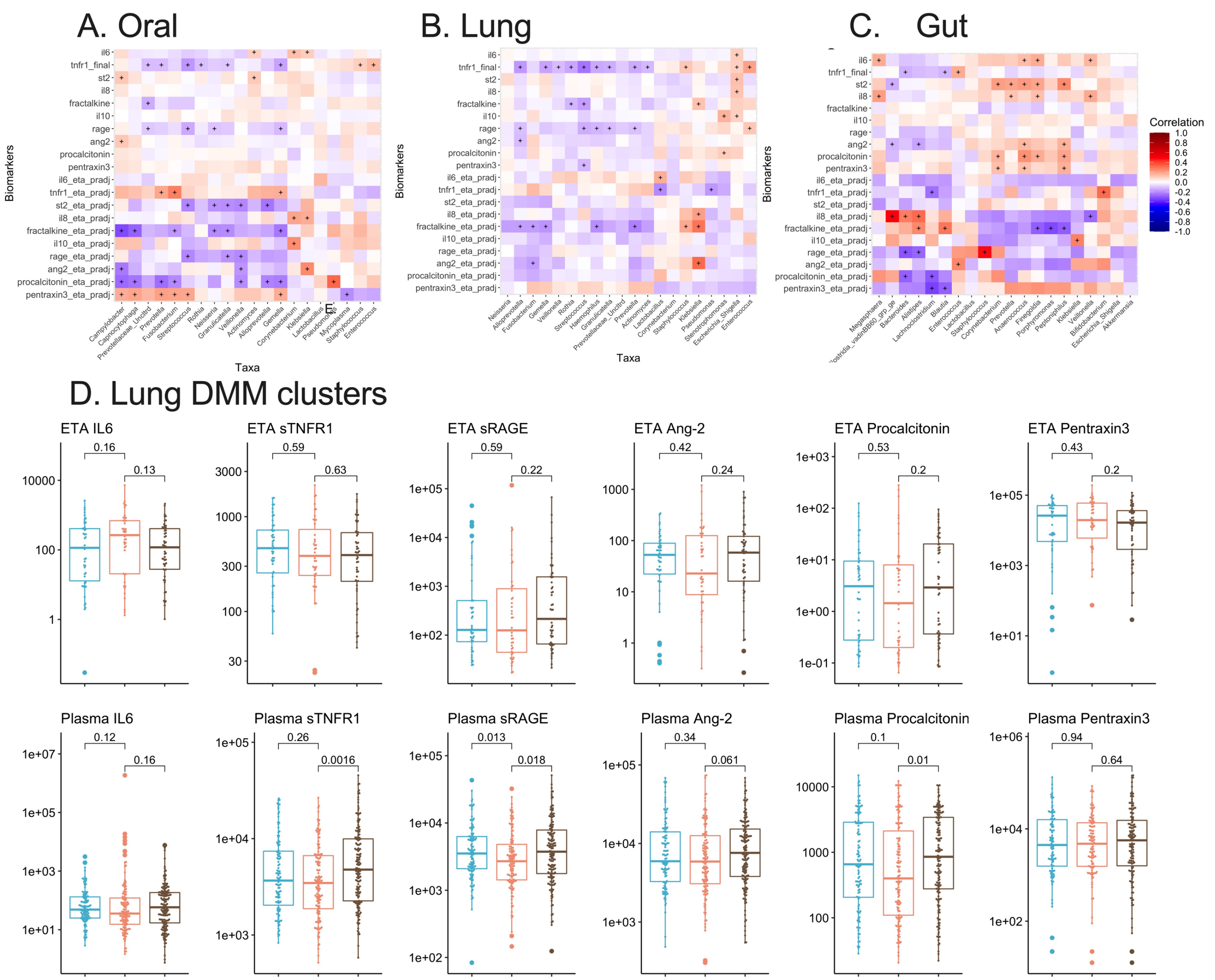

Microbiota correlate with host response biomarkers at both the lung compartment and at a systemic level. A-C: Heatmaps of correlations between the top 20 abundant taxa in the oral (A), lung (B) and gut (C) compartment with 10 host response biomarkers measured in plasma samples (top 10 rows) and endotracheal aspirate (ETA) supernatant samples (bottom 10 rows) in each heatmap. ETA biomarker values were adjusted for total protein concentration in each sample. Statistically significant correlations adjusted for multiple testing (Benjamini-Hochberg method) are shown with crosses ("+") and the direction of the correlation is color coded. D: Comparisons of ETA and plasma biomarkers between bacterial DMM clusters. The low diversity bacterial DMM cluster (brown) had significantly higher levels of plasma sTNFR1, sRAGE and procalcitonin levels. Source data are provided as a Source Data file. Data displayed as boxplots with individual dots have their

median as the line inside the box, interquartile range (25th-75th percentile) as the box itself, whiskers extend to 1.5 times the interquartile range, and individual dots beyond whiskers signify outlier observations.

Figure S10:

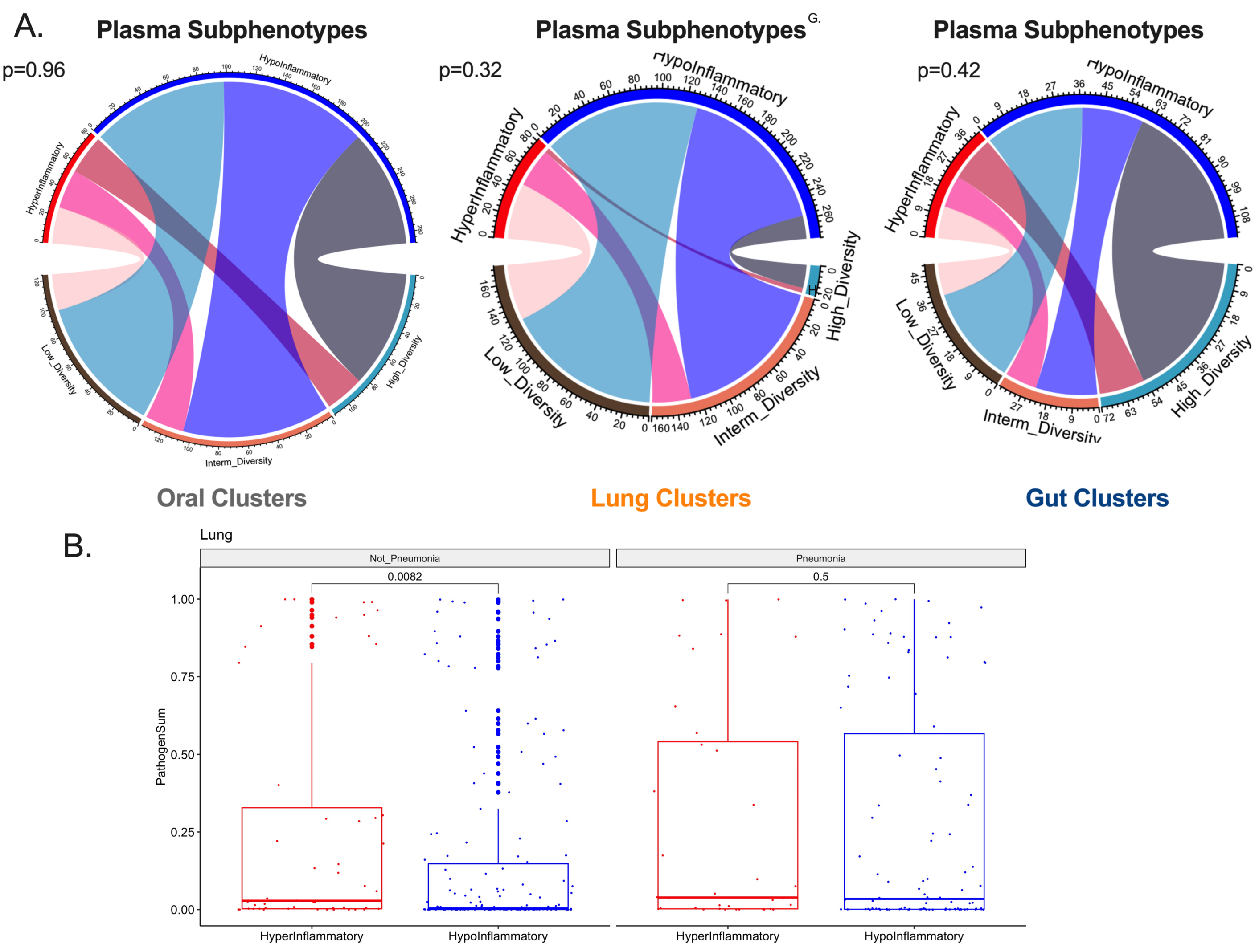

Host-centric approach for relationships between microbiota and host responses. A. Chord plots for plasma-derived subphenotypes of host response (hyper- vs. hypo-inflammatory as predicted by a 4-variable regression model) and bacterial DMM clusters. No significant associations were found. H. Hyper-inflammatory patients had higher abundance of pathogens in lung samples compared to hypo-inflammatory patients, driven by the subset of patients without diagnosis of pneumonia. Source data are provided as a Source Data file. Data displayed as boxplots with individual dots have their median as the line inside the box, interquartile range (25th-75th percentile) as the box itself, whiskers extend to 1.5 times the interquartile range, and individual dots beyond whiskers signify outlier observations.

Figure S11.

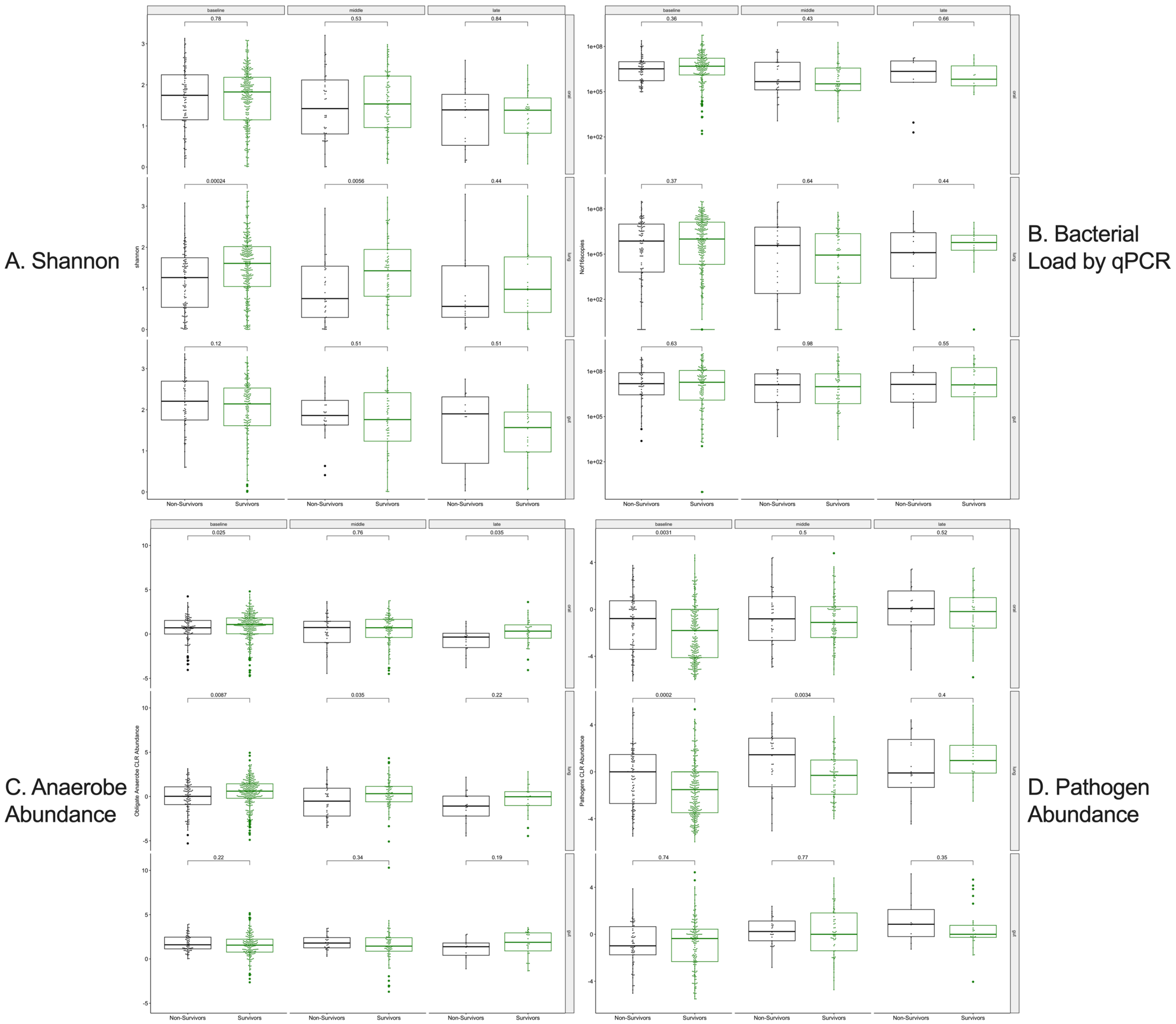

Microbiota differences between 60-day survivors and non-survivors in the oral, lung and gut compartments. Serial differences in Shannon index (A), bacterial load by 16S qPCR (B), obligate anaerobe abundance (C) and pathogen abundance (D) between survivors and non-survivors in each compartment. Relative abundances were CLR-transformed. In the baseline and middle interval, survivors had higher Shannon index, higher abundance of anaerobes and lower abundance of pathogens in the lung compartment. In the baseline interval, survivors had higher abundance of anaerobes and lower abundance of pathogens in the oral compartment. Source data are provided as a Source Data file. Data displayed as boxplots with

individual dots have their median as the line inside the box, interquartile range (25th-75th percentile) as the box itself, whiskers extend to 1.5 times the interquartile range, and individual dots beyond whiskers signify outlier observations.

Figure S12:

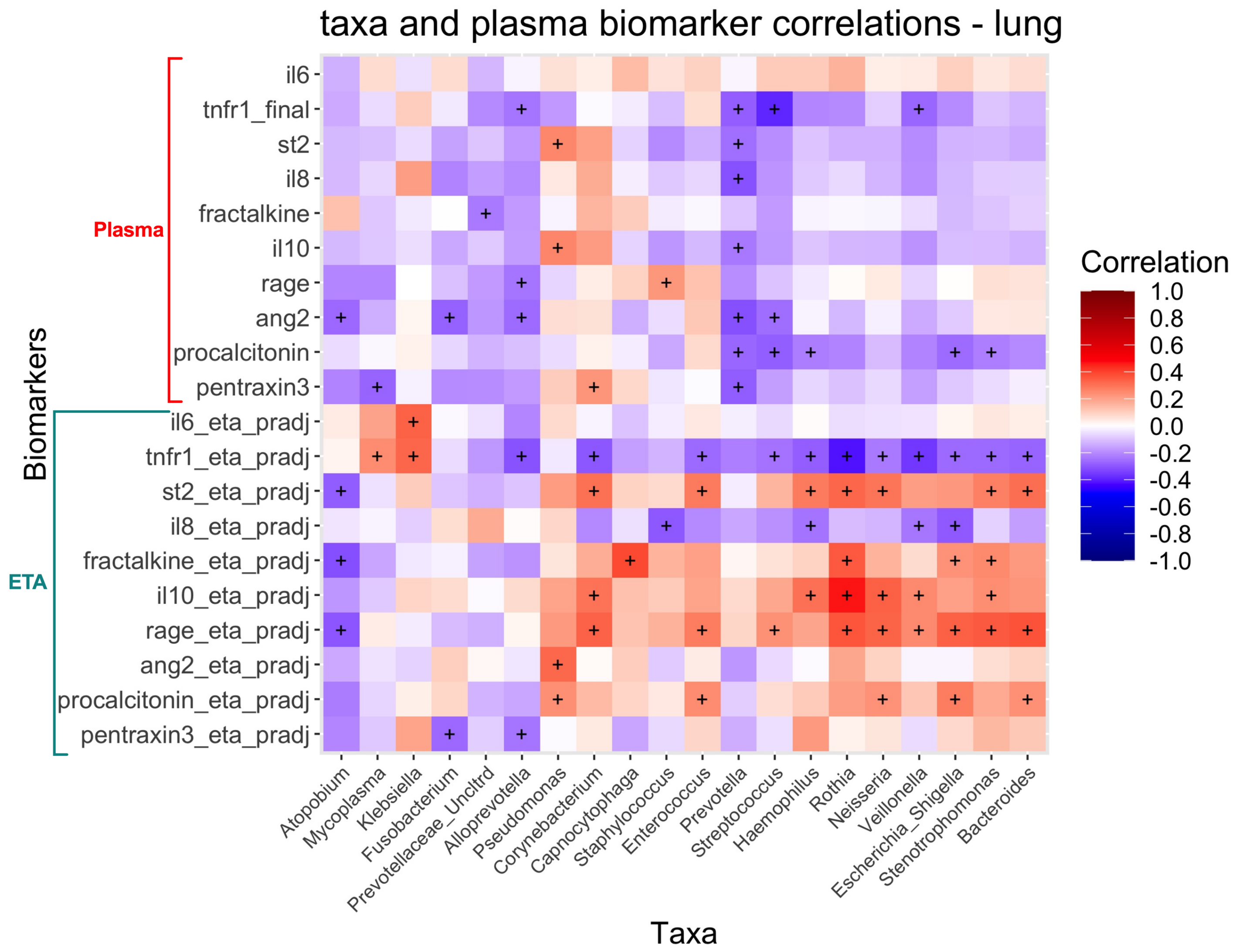

Lung bacteria correlate with host response biomarkers at both the lung compartment and at a systemic level in patients with severe COVID-19. Heatmap of correlations between the top 20 abundant taxa in lung samples with 10 host response biomarkers measured in plasma samples (top 10 rows) and endotracheal aspirate (ETA) supernatant samples (bottom 10 rows) in each heatmap. ETA biomarker values were adjusted for total protein concentration in each sample. Statistically significant correlations adjusted for multiple testing (Benjamini-Hochberg method) are shown with crosses ("+") and the direction of the correlation is color coded. Source data are provided as a Source Data file.
