# Supplementary material for: Longitudinal multicompartment characterization of host-microbiota interactions in patients with acute respiratory failure
Source: Nat Commun. 2024 Jun 3;15:4708. doi: 10.1038/s41467-024-48819-8 (PMC11148165; doi:10.1038/s41467-024-48819-8)
Supplement: Supplementary file 3 — Reporting Summary [file 41467_2024_48819_MOESM3_ESM.pdf]

nature portfolio

Corresponding author(s): Georgios Kitsios

Last updated by author(s): May 3, 2024

Reporting Summary

Nature Portfolio wishes to improve the reproducibility of the work that we publish. This form provides structure for consistency and transparency in reporting. For further information on Nature Portfolio policies, see our Editorial Policies and the Editorial Policy Checklist.

Statistics

For all statistical analyses, confirm that the following items are present in the figure legend, table legend, main text, or Methods section.

n/a	Confirmed	
☐	☒	The exact sample size (*n*) for each experimental group/condition, given as a discrete number and unit of measurement
☐	☒	A statement on whether measurements were taken from distinct samples or whether the same sample was measured repeatedly
☐	☒	The statistical test(s) used AND whether they are one- or two-sided<br>*Only common tests should be described solely by name; describe more complex techniques in the Methods section.*
☐	☒	A description of all covariates tested
☐	☒	A description of any assumptions or corrections, such as tests of normality and adjustment for multiple comparisons
☐	☒	A full description of the statistical parameters including central tendency (e.g. means) or other basic estimates (e.g. regression coefficient) AND variation (e.g. standard deviation) or associated estimates of uncertainty (e.g. confidence intervals)
☐	☒	For null hypothesis testing, the test statistic (e.g. *F*, *t*, *r*) with confidence intervals, effect sizes, degrees of freedom and *P* value noted<br>*Give P values as exact values whenever suitable.*
☐	☒	For Bayesian analysis, information on the choice of priors and Markov chain Monte Carlo settings
☐	☒	For hierarchical and complex designs, identification of the appropriate level for tests and full reporting of outcomes
☐	☒	Estimates of effect sizes (e.g. Cohen's *d*, Pearson's *r*), indicating how they were calculated

Our web collection on statistics for biologists contains articles on many of the points above.

Software and code

Policy information about availability of computer code

Data collection	We obtained clinical data directly from the electronic medical record. Molecular assay data were obtained in the research lab with the assays reported in the Methods. Raw sequencing data have been deposited in public repositories. We analyzed data with available R packages.
Data analysis	The custom R code used for these analysis is available on the GitHub Repository and linked to a Zenodo DOI. Primary code is available on the GitHub repository (https://github.com/MicrobiomeALIR/MultiCompartmentMicrobiome), with an archive of the code including a Digital Object Identifier available through Zenodo at 10.5281/zenodo.11109543.<br>We used R version 4.2.0, KneadData 0.10.0, MetaPhlAn 3.0.0, Mothur-based v1.44.1

For manuscripts utilizing custom algorithms or software that are central to the research but not yet described in published literature, software must be made available to editors and reviewers. We strongly encourage code deposition in a community repository (e.g. GitHub). See the Nature Portfolio guidelines for submitting code & software for further information.

Data

Policy information about availability of data

All manuscripts must include a data availability statement. This statement should provide the following information, where applicable:

- Accession codes, unique identifiers, or web links for publicly available datasets
- A description of any restrictions on data availability
- For clinical datasets or third party data, please ensure that the statement adheres to our policy

The Sequencing data collected for the study have been deposited to the Sequencing Resource Archive, through the following Accession numbers:
-PRJNA595346 for 16S data of UPMC-ARF and UPMC-COVID cohorts (477 records released and remainder to be released upon publication with Temporary Submission ID SUB13319619), available at https://www.ncbi.nlm.nih.gov/bioproject/PRJNA595346
-PRJNA726955 for ITS data of UPMC-ARF cohort, available at: https://www.ncbi.nlm.nih.gov/bioproject/726955
-PRJNA554461 for Nanopore data of UPMC-ARF cohort (71 records released and remainder to be released upon publication with Temporary Submission IDs SUB14129477 and SUB14129116), available at: https://www.ncbi.nlm.nih.gov/bioproject/PRJNA554461
-PRJNA940725 for 16S data of the Healthy Controls, available at https://www.ncbi.nlm.nih.gov/bioproject/PRJNA940725
-PRJNA976404 for Metagenomic data of the MGH-COVID cohort, available at https://www.ncbi.nlm.nih.gov/bioproject/PRJNA976404
De-identified clinical and processed microbiome data for the replication of analyses are available on the GitHub repository (https://github.com/MicrobiomeALIR/MultiCompartmentMicrobiome).
Source data are provided with this paper for all figures and Tables.

Research involving human participants, their data, or biological material

Policy information about studies with human participants or human data. See also policy information about sex, gender (identity/presentation), and sexual orientation and race, ethnicity and racism.

Reporting on sex and gender	We obtained clinical data directly from the electronic medical record. We captured biological sex and race as recorded in the medical record.
Reporting on race, ethnicity, or other socially relevant groupings	We obtained clinical data directly from the electronic medical record. We captured biological sex and race as recorded in the medical record.
Population characteristics	Patients had a median (interquartile range) age of 59.6 (46.7-68.7) years, 54.4% were men and 90.2% were white (Table 1). At the time of enrollment, 25.0% of patients were diagnosed with Acute Respiratory Distress Syndrome (ARDS per the Berlin definition14) and 39.8% with pneumonia, 86.8% were receiving systemic antibiotics, and 64.8% received corticosteroids for various indications. By 60 days, 26.9% of patients had died. Among the 350 patients who survived hospitalization, 48.8% were discharged to their home, with the remainder requiring additional longer-term care
Recruitment	We recruited hospitalized patients with acute respiratory failure from COVID and nonCOVID etiologies.
Ethics oversight	Ethics approval and consent to participate: The University of Pittsburgh Institutional Review Board (IRB) approved the protocol for the UPMC-ARF and UPMC-COVID cohorts (STUDY19050099). We obtained written or electronic informed consent by all participants or their surrogates in accordance with the Declaration of Helsinki. For the MGH-COVID cohort, the Study protocol #2020P000804 was approved by the Mass General Brigham IRB. For the healthy controls, the University of Pittsburgh IRB approved the study protocols (STUDY19060243 for respiratory biospecimens and STUDY20060312 for stool biospecimens). All participants or their healthcare proxy provided written informed consent to participate.

Note that full information on the approval of the study protocol must also be provided in the manuscript.

Field-specific reporting

Please select the one below that is the best fit for your research. If you are not sure, read the appropriate sections before making your selection.

- [x] Life sciences
- [ ] Behavioural & social sciences
- [ ] Ecological, evolutionary & environmental sciences

For a reference copy of the document with all sections, see nature.com/documents/nr-reporting-summary-flat.pdf

Life sciences study design

All studies must disclose on these points even when the disclosure is negative.

Sample size	We used feasibility datasets by recruiting patients with acute respiratory failure from 2015-2022 and report the largest, to our knowledge, ICU cohort with microbiome analysis.
Data exclusions	We considered clinical samples that generated ≥1,000 quality 16S-Seq reads. Clinical samples that generated fewer than 1,000 reads were not considered of sufficient sequencing yield to be acceptable for analysis.
Replication	We validated our main findings in two independent cohorts (MGH-COVID and UPMC-COVID).

April 2023

Randomization	Our study was observational and not randomized.
Blinding	A consensus committee reviewed clinical and radiographic data and performed retrospective classifications of the etiology and severity of acute respiratory failure without knowledge of microbiome sequencing or biomarker data

Reporting for specific materials, systems and methods

We require information from authors about some types of materials, experimental systems and methods used in many studies. Here, indicate whether each material, system or method listed is relevant to your study. If you are not sure if a list item applies to your research, read the appropriate section before selecting a response.

Materials & experimental systems

n/a	Involved in the study
☒	☐ Antibodies
☒	☐ Eukaryotic cell lines
☒	☐ Palaeontology and archaeology
☒	☐ Animals and other organisms
☐	☒ Clinical data
☒	☐ Dual use research of concern
☒	☐ Plants

Methods

n/a	Involved in the study
☒	☐ ChIP-seq
☒	☐ Flow cytometry
☒	☐ MRI-based neuroimaging

Clinical data

Policy information about clinical studies

All manuscripts should comply with the ICMJEguidelines for publication of clinical research and a completedCONSORT checklist must be included with all submissions.

Clinical trial registration	Our study was observational and not randomized
Study protocol	We have reported our methods previously and provide citations to our prior work. Our study was not randomized.
Data collection	In the UPMC-ARF cohort, we enrolled patients with non-COVID etiologies of ARF between March 2015 and June 2022. We collected baseline research biospecimens within 72hrs from intubation, including blood for separation of plasma, oropharyngeal swabs (oral samples), endotracheal aspirates (ETA) collected for research or excess bronchoalveolar lavage fluid (BALF) from clinical bronchoscopy (lung samples), and rectal swabs or stool (gut samples).3,11,12 We repeated research biospecimen sampling between days 3-6 (middle interval) and days 7-12 (late interval) post-enrollment for subjects who remained in the ICU
Outcomes	We followed patients prospectively for cumulative mortality and ventilator-free days (VFDs) at 30 days, as well as survival up to 60 days from intubation

Plants

Seed stocks	*Report on the source of all seed stocks or other plant material used. If applicable, state the seed stock centre and catalogue number. If plant specimens were collected from the field, describe the collection location, date and sampling procedures.*
Novel plant genotypes	*Describe the methods by which all novel plant genotypes were produced. This includes those generated by transgenic approaches, gene editing, chemical/radiation-based mutagenesis and hybridization. For transgenic lines, describe the transformation method, the number of independent lines analyzed and the generation upon which experiments were performed. For gene-edited lines, describe the editor used, the endogenous sequence targeted for editing, the targeting guide RNA sequence (if applicable) and how the editor was applied.*
Authentication	*Describe any authentication procedures for each seed stock used or novel genotype generated. Describe any experiments used to assess the effect of a mutation and, where applicable, how potential secondary effects (e.g. second site T-DNA insertions, mosiacism, off-target gene editing) were examined.*
